# Hyaluronan Does Not Affect Bupivacaine's Inhibitory Action on Voltage-Gated Potassium Channel Activities in Bovine Articular Chondrocytes

**DOI:** 10.1155/2012/361534

**Published:** 2012-04-22

**Authors:** William Hester, Jinnan Yang, Guo-Yong Wang, Sen Liu, Michael J. O'Brien, Felix H. Savoie, Zongbing You

**Affiliations:** ^1^Department of Structural and Cellular Biology, Tulane University School of Medicine, New Orleans, LA 70112, USA; ^2^Department of Orthopaedic Surgery and Tulane Institute of Sports Medicine, Tulane University School of Medicine, New Orleans, LA, USA; ^3^Tulane Cancer Center, Louisiana Cancer Research Consortium, Tulane Center for Aging, Tulane Center for Stem Cell Research and Regenerative Medicine, Tulane University School of Medicine, New Orleans, LA 70112, USA

## Abstract

*Objectives.* The objective of this paper is to determine if hyaluronan affects bupivacaine's anesthetic function. *Methods.* Whole cell patch clamp recordings were performed on bovine articular chondrocytes cultured in 60 mm dishes. The chondrocytes were treated with phosphate-buffered saline (control group), 7.5 mg/mL hyaluronan (Orthovisc), 0.25% bupivacaine, or a mixture of 7.5 mg/mL hyaluronan and 0.25% bupivacaine. Outward currents were elicited by step depolarization from −90 mV to 150 mV with 5 mV increments and holding for 200 ms. *Results.* The amplitude of outward currents elicited at 150 mV was 607.1 ± 135.4 pA (mean ± standard error) in the chondrocytes treated with phosphate buffered saline, 550.0 ± 194.9 pA in the chondrocytes treated with hyaluronan, 18.4 ± 8.3 pA in the chondrocytes treated with bupivacaine, and 12.8 ± 2.6 pA in the chondrocytes treated with a mixture of hyaluronan and bupivacaine. *Conclusion.* Hyaluronan does not affect bupivacaine's inhibitory action on the potassium channel activities in bovine articular chondrocytes. This finding suggests that intra-articular injection of a mixture of hyaluronan and bupivacaine may not affect the anesthetic effects of bupivacaine.

## 1. Introduction

Bupivacaine is a local anesthetic that is an amine compound and is commonly used after arthroscopic surgery for postoperative pain control [1]. Bupivacaine achieves its anesthetic effect by binding to sodium, potassium, and calcium channels of neurons thereby inhibiting propagation of action potentials [2, 3]. Scholz reported that the receptor for bupivacaine is localized inside the channels, not on the extracellular surface of them [2]. Orthovisc is a bacterially derived, high molecular weight (1.0–2.9 million daltons) sodium hyaluronate dissolved in a physiologic saline solution with a hyaluronan concentration of 15 mg/mL (manufactured by Anika Therapeutics, Inc., Woburn, Massachusetts, and distributed by Depuy Mitek, Raynham, Massachusetts). Orthovisc is indicated for the treatment of arthritic pain in human knee joints [4]. Gomis et al. proposed that the highly elastoviscous hyaluronan solutions achieved their anesthetic effect by reducing the transmission of signals to stretch activated channels in nociceptive nerve endings [5]. This implies that hyaluronan has a different mechanism of anesthetic action than bupivacaine.

Several recent *in vitro* studies have shown that bupivacaine can cause reduced chondrocyte function and even chondrocyte death [6–8]. Some studies have shown that intra-articular injection of bupivacaine may cause chondrolysis particularly when administered via intra-articular infusion pumps [9, 10]. We have recently reported that hyaluronan may prevent bupivacaine-induced chondrotoxicity in bovine articular chondrocytes exposed to bupivacaine at supraphysiologic temperatures [11]. This finding hints at a potential of intra-articular injection of a mixture of bupivacaine and hyaluronan; thus bupivacaine's toxicity may be prevented by hyaluronan. However, it is not known whether hyaluronan may also inhibit bupivacaine's anesthetic effects. Thus, we conducted this study to determine whether hyaluronan affects bupivacaine's action on the voltage-gated potassium channels of bovine articular chondrocytes using whole cell patch clamp recordings.

## 2. Materials and Methods

### 2.1. Cell Culture

Normal bovine articular chondrocytes were harvested from the stifle joints (equivalent to human knee joints) of 3-week-old calves obtained from an abattoir. The chondrocytes were cultured with Dulbecco's Modified Eagle's Medium (DMEM) containing 10% fetal bovine serum and 1% penicillin/streptomycin (Invitrogen, Carlsbad, California) in a 5% CO_2_ humidified incubator at 37°C. The cells used in this study were cultured in a monolayer for less than three weeks.

### 2.2. Treatment of Cells in Monolayer Culture

In order to assess the effects of bupivacaine (Hospira, Inc., Lake Forest, Illinois) and hyaluronan (Orthovisc) on bovine articular chondrocytes, the cells were plated in 60 mm dishes at a density of 15,000 cells per dish and incubated at 37°C in a 5% CO_2_-humidified incubator prior to treatment. The medium was siphoned off, and the monolayer cultures were rinsed twice with 3 mL of phosphate buffered saline (PBS). The cells were then treated with PBS or the treatment solutions per the protocol below at room temperature. The treated cells were allowed to incubate at room temperature for at least ten minutes but no longer than ninety minutes before patch clamp recordings were completed.

The treatment groups included the following:

1. control group: treated with PBS,
2. bupivacaine group: treated with 0.25% bupivacaine in PBS,
3. hyaluronan group: treated with 50% Orthovisc in PBS (containing 7.5 mg/mL hyaluronan),
4. Hyaluronan/bupivacaine group: treated with a combination of 7.5 mg/mL hyaluronan and 0.25% bupivacaine.

### 2.3. Whole Cell Patch Clamp Recording

The following procedure was based on a recently published whole cell patch clamp technique [12]. Patch pipettes were pulled from thick walled 1.5 mm borosilicate glass with a tip resistance between 3 and 7 MΩ on an automatic micropipette puller (Sutter Instruments, model P-97, Novato, California). The micropipette served as a probe with an electrode connected to the MultiClamp 700B patch clamp amplifier (Molecular Devices, Sunnyvale, California). Live cells attached to the culture plate were identified using light microscopy at 100x magnification (Nikon Eclipse Microscope, Japan), and then the patch pipette was placed approximately over the cell. Using 400x magnification, the pipette was lowered onto the chondrocyte cell surface, and gentle suction was applied to create a high-resistance seal. Transient currents caused by pipette capacitance were electronically compensated by the circuit of the amplifier. If the seal resistance reached >1 GΩ, then recordings were made. The series resistance was 7–16 MΩ, and recordings were discarded if significant increases (>20%) in series resistance occurred. Whole cell patch recordings were then made using the MultiClamp 700 B amplifier. Inward and outward currents were recorded at membrane potentials ranging from −90 mV to 150 mV with 5 mV steps lasting 200 ms. After the recordings were made, resting membrane potentials were read from the amplifier. The data were lowpass filtered at 2 kHz and digitized at rates of 5 kHz before they were stored on the computer for later analysis. The amplitudes of the outward current (pA) were recorded before and after drug treatment.

### 2.4. Statistical Methods

Each cell was recorded three times, and five cells were recorded per treatment group (*n* = 5). The means and standard errors (SEs) were compared between the PBS control group and each treatment group using the two-tailed Student's *t*-test. The level of significance was set at *P* < 0.05.

## 3. Results

**Figure 1:**
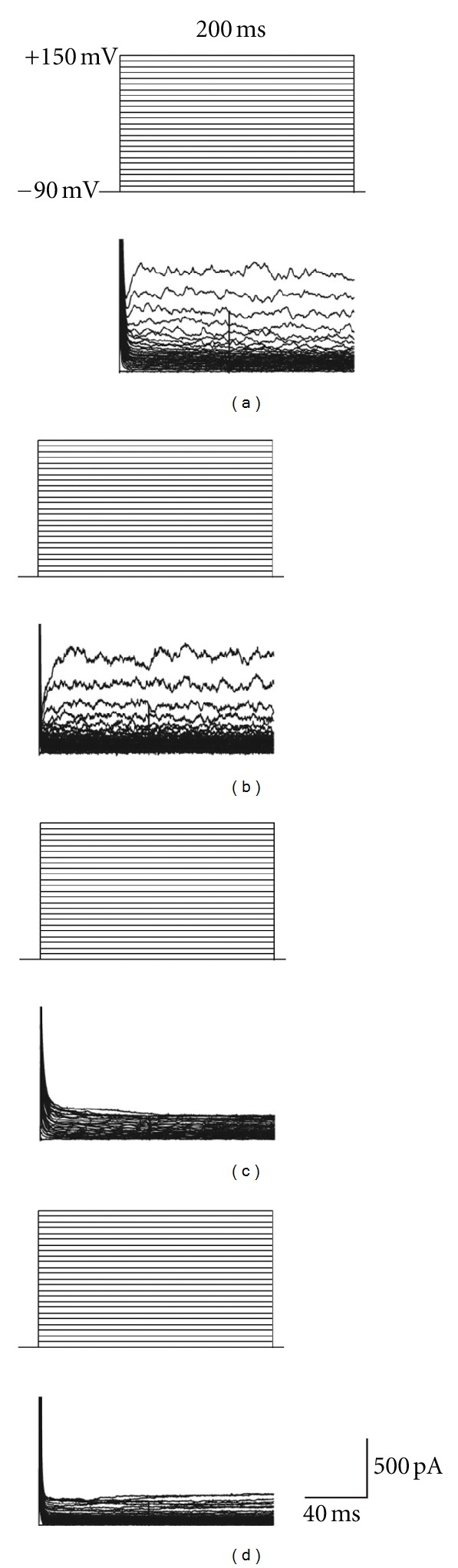
Representative whole cell patch clamp recordings showing outward currents elicited by step depolarization from −90 mV to 150 mV with 5 mV increments and holding for 200 ms. Bovine articular chondrocytes were treated with PBS (a), 7.5 mg/mL hyaluronan (b), 0.25% bupivacaine (c), and a mixture of 7.5 mg/mL hyaluronan and 0.25% bupivacaine (d).

Patch electrodes were initially filled with a solution containing 118 mM cesium methane sulfonate, 12 mM cesium chloride, 0.5 mM calcium chloride, 0.5 mM magnesium chloride, 10 mM 4-(2-hydroxyethyl)-1-piperazineethanesulfonic acid (HEPES), and 5 mM ethylene glycol tetraacetic acid (EGTA), with an osmolarity of 290 and pH of 7.3. After successfully obtaining a seal on 5 different chondrocytes, no inward or outward currents were able to be elicited. This prompted us to use a different solution thereby allowing the study of outward currents that were found at membrane potentials from 140 mV to 150 mV. Patch electrodes were then filled with a solution containing 120 mM potassium gluconate, 10 mM sodium chloride, 0.5 mM magnesium chloride, 0.5 mM calcium chloride, 10 mM HEPES, and 5 mM EGDA, with an osmolarity of 290 and pH of 7.3. From a holding potential of −90 mV depolarized to 150 mV, outward currents were elicited in chondrocytes treated with PBS (Figure 1(a)) and hyaluronan (Figure 1(b)). In contrast, the outward currents were completely inhibited when the chondrocytes were treated with bupivacaine (Figure 1(c)) or a combination of bupivacaine and hyaluronan (Figure 1(d)).

**Figure 2:**
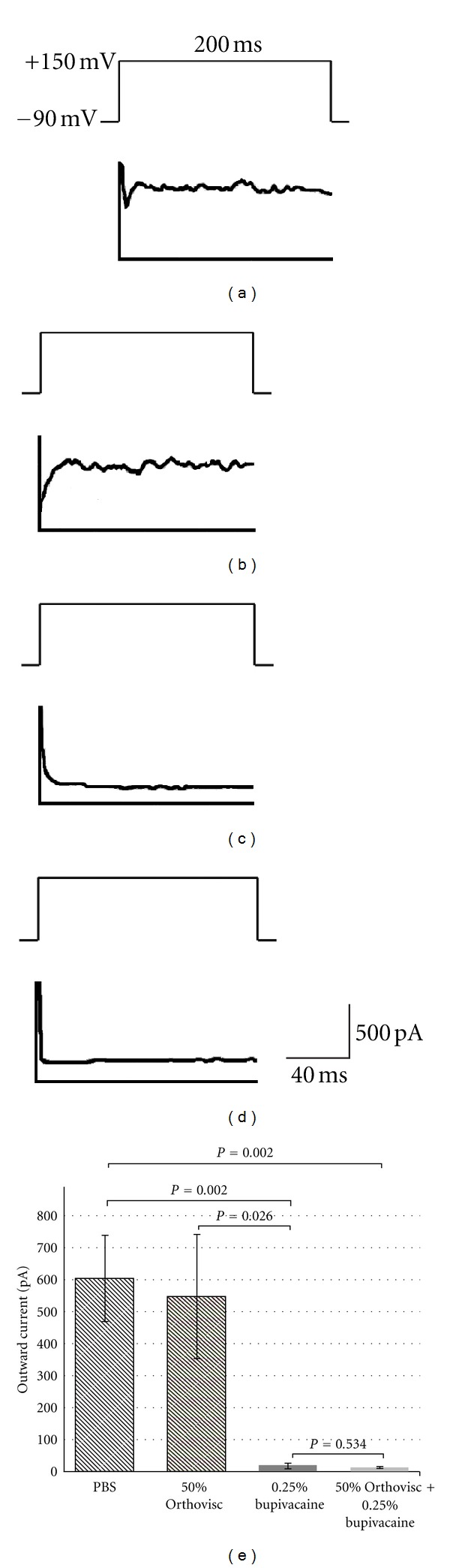
Representative whole cell patch clamp recordings showing outward currents elicited by depolarization at 150 mV with holding for 200 ms. Bovine articular chondrocytes were treated with PBS (a), 7.5 mg/mL hyaluronan (b), 0.25% bupivacaine (c), and a mixture of 7.5 mg/mL hyaluronan and 0.25% bupivacaine (d). (e) Means and standard errors (error bars) of the outward currents recorded in five chondrocytes (*n* = 5). *P* values were obtained by Student's *t*-test (two-tailed).

In order to analyze the data quantitatively, we examined the amplitudes of outward currents elicited at 150 mV (Figures 2(a) to 2(d)). When the cells were treated with PBS (as control), the mean outward current was 607.1 pA (SE = 135.4) (Table 1 and Figure 2(e)). When the cells were treated with hyaluronan, the mean outward current was 550.0 pA (SE = 194.9) (Table 1 and Figure 2(e)). There was no significant difference in the outward currents between the control group and hyaluronan treatment group (*P* = 0.816). When the cells were treated with 0.25% bupivacaine, the mean outward current decreased to 18.4 pA (SE = 8.3), which was significantly different from the control and hyaluronan treatment groups (*P* = 0.002 and *P* = 0.026, resp.) (Table 1 and Figure 2(e)). When the cells were treated with a combination of bupivacaine and hyaluronan, the mean outward current was 12.8 pA (SE = 2.6), which was significantly different from the control group (*P* = 0.002) (Table 1 and Figure 2(e)). There was no significant difference between bupivacaine alone group and hyaluronan/bupivacaine combined group (*P* = 0.534).

**Figure 3:**
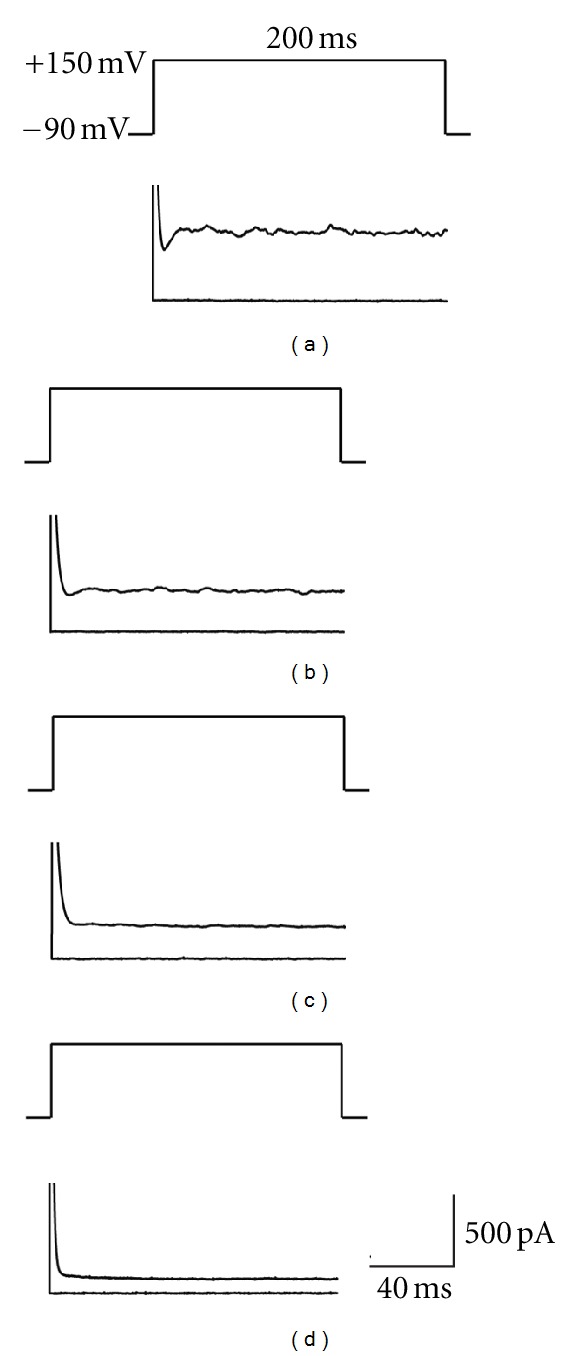
Representative whole cell patch clamp recordings showing outward currents elicited at 150 mV with holding for 200 ms. Bovine articular chondrocytes were treated with PBS (a), 0.25% bupivacaine for 5 minutes (b), 0.25% bupivacaine for 10 minutes (c), and cesium (potassium channel blocker) (d).

To make sure that we were testing live cells in the bupivacaine treated group, we examined a live cell that had been treated with PBS and recorded a mean outward current at 150 mV of 491.4 pA (SE = 113.3) (Figure 3(a)). Once the recordings were obtained, we treated the cell with 0.25% bupivacaine. Then, we took recordings at five and ten minutes after bupivacaine treatment on the same cell at 150 mV. Five minutes after bupivacaine treatment, we recorded a mean outward current of 190.8 pA (SE = 35.4) (Figure 3(b)); ten minutes after bupivacaine treatment, we recorded a mean outward current of 13.1 pA (SE = 2.8) (Figure 3(c)). When we added cesium (a potassium channel blocker) to the micropipette solution, the outward currents were completely blocked (Figure 3(d)).

## 4. Discussion

We performed this study to determine if hyaluronan can influence the anesthetic action of bupivacaine. Bupivacaine acts on sodium, potassium, and calcium channels of neuron cells to inhibit pain sensation. Because we could not find a source of normal neuron cell line and it was difficult to isolate any primary neurons for our study, we used bovine articular chondrocytes. We believe that this is appropriate because bupivacaine acts on channel proteins that are the same in both chondrocytes and neurons. Initially, we planned to study the effects of bupivacaine on the voltage-gated sodium channels as this is the primary mechanism of local anesthetics with an amine group [2]. However, we were unable to elicit any inward currents in bovine articular chondrocytes. We were able to elicit outward currents that were inhibited by cesium, a known potassium channel blocker [13], suggesting that the outward currents were caused by potassium channel activities. It has been reported that bupivacaine can block both sodium and potassium channels [2, 14, 15]. Therefore, our study focused on the effects of bupivacaine on potassium channel activities.

We have previously shown that bupivacaine does not cause chondrocyte death when bovine articular chondrocytes were treated with bupivacaine in PBS at room temperature [16]. To make sure that the chondrocyte was alive, we first recorded the outward currents when the cell was treated with PBS and then switched the treatment to bupivacaine. We observed a gradual decrease of the amplitudes of the outward currents after the chondrocyte was treated with bupivacaine for 5 to 10 minutes. This suggests that the decrease of outward currents is caused by bupivacaine's pharmacologic action on the potassium channels, rather than death of the chondrocyte.

In the present study, we found that hyaluronan had no effects on the voltage-gated potassium channel activities. In contrast, bupivacaine completely inhibited the potassium channel activities. Moreover, hyaluronan did not increase or decrease bupivacaine's inhibition on the potassium channel activities when hyaluronan was mixed with bupivacaine. These findings suggest that hyaluronan, if mixed with bupivacaine, may not affect bupivacaine's anesthetic function. It has been reported in animal [17] and human studies [18] that the duration of sensory nerve blockade from bupivacaine is prolonged by addition of hyaluronan. Hassan et al. described this extended action of the local anesthetic, but they did not determine the exact mechanism. They speculated that hyaluronan may bind to the local anesthetic drug, thereby delaying the drug's access to the channel proteins [17]. We have previously shown that hyaluronan can prevent bupivacaine-induced chondrotoxicity [11]. Grishko et al. demonstrated that chondrotoxicity is accompanied by mitochondrial dysfunction leading to deficient energy production and cell death when the cells were exposed to local anesthetics [19]. However, hyaluronan can prevent chondrocyte death by decreasing mitochondrial DNA damage, enhancing mitochondrial DNA repair capacity, and preserving cellular ATP levels [20]. The findings from the present study demonstrate that hyaluronan may not affect bupivacaine's anesthetic function. Taken together, it is reasonable to speculate that intra-articular injection of a mixture of hyaluronan and bupivacaine may offer adequate pain relief and avoid potential side effects caused by bupivacaine.

## 5. Conclusion

Hyaluronan does not affect bupivacaine's inhibitory action on the potassium channel activities in bovine articular chondrocytes. This finding suggests that intra-articular injection of a mixture of hyaluronan and bupivacaine may not affect the anesthetic effects of bupivacaine.

## Figures and Tables

**Table 1:** Outward current (pA) elicited at 150 mV in bovine articular chondrocytes.

	PBS	Hyaluronan	Bupivacaine	Hyaluronan/Bupivacaine
Cell 1	454.5	297.6	13.1	6.8
Cell 2	1083.5	446.3	51.1	14.0
Cell 3	536.1	1313.0	13.6	11.3
Cell 4	679.8	246.6	8.7	9.8
Cell 5	281.4	446.5	5.6	21.9
*Mean*	**607.1**	**550.0**	**18.4**	**12.8**
*Standard Error*	**135.4**	**194.9**	**8.3**	**2.6**
*P* (versus PBS)		0.816	0.002	0.002
*P* (versus Hyaluronan)			0.026	0.025
*P* (versus Bupivacaine)				0.534
